#  Adverse events of capecitabine in gastric cancer patients: a real‐world pharmacovigilance study based on the FAERS database

**DOI:** 10.3389/fphar.2026.1861329

**Published:** 2026-09-11

**Authors:** Ru Sang, Mingxia Zhu, Jibin Mao, Yi Wu, Songbing Qin, Lili Wang

**Affiliations:** Department of Radiation Oncology, The First Affiliated Hospital of Soochow University, Suzhou, China

**Keywords:** adverse event, capecitabine, FAERS, gastric cancer, pharmacovigilance

## Abstract

**Background:**

Capecitabine stands as a pivotal chemotherapeutic agent in gastric cancer (GC) management. While its general safety profile has been documented, comparative pharmacovigilance analyses of distinct capecitabine-based treatment regimens among GC patients remain scarce.

**Methods:**

This study extracted AE reports from the United States Food and Drug Administration AE Reporting System (FAERS) for the period 2004 Q1-2024 Q4. Following rigorous data cleansing, the adverse event (AE) reports were systematically standardized using the system organ class (SOC) and preferred term (PT) terminology from the Medical Dictionary for Regulatory Activities (MedDRA, version 26.0). Four disproportionality algorithms were employed to detect and analyze AE signals across four cohorts. Cohort 1 was from capecitabine monotherapy. Cohort 2 represented capecitabine in combination with other chemotherapeutic agents. Cohort 3 was for capecitabine in combination with other chemotherapeutic and targeted agents. Cohort 4 was from the capecitabine in combination with other chemotherapeutic agents as well as immunosuppressants.

**Results:**

A total of 1,681 AE reports associated with capecitabine were included (632 cases in Cohort 1, 816 cases in Cohort 2, 189 cases in Cohort 3, and 44 cases in Cohort 4). The outcomes of AEs were predominantly other medical events, hospitalization or prolonged hospitalization, and death. Gastrointestinal disorders, general disorders and administration site conditions were the most frequently affected SOCs. The most frequent AEs in Cohort 1 were death (n = 83), diarrhea (n = 75), and nausea (n = 73), whereas the strongest signal was palmar-plantar erythrodysaesthesia syndrome (n = 43, ROR = 4.62). In Cohort 2, diarrhoea (n = 160), vomiting (n = 146), and nausea (n = 143) were the most frequently reported AEs, whereas palmar-plantar erythrodysaesthesia syndrome showed the strongest signal (n = 95, ROR = 6.69). In Cohort 3, leukoencephalopathy exhibited the strongest signal (n = 7, ROR = 21.79). In Cohort 4, sleep disorder (n = 5, ROR = 95.98, 95% CI: 33.54–274.69), dry mouth (n = 5, ROR = 35.98, 95% CI: 13.89–93.18), and inappropriate schedule of product administration (n = 5, ROR = 28.78, 95% CI: 11.25–73.59) appeared as novel, robust signals.

**Conclusion:**

Capecitabine showed distinct AE profiles in different GC regimens, with both prevalent and rare signals. Tailored safety monitoring is needed based on combination regimens.

## 1 Introduction

Capecitabine is an oral prodrug of fluorouracil, serving as a cornerstone chemotherapeutic agent in various solid tumors (Wang et al., 2021; Tomao et al., 2021; Kunz et al., 2023). The active metabolite 5-fluorouracil (5-FU) irreversibly inhibits thymidylate synthase (TS), thereby disrupting synthesis and replication in DNA (Matuszyk, 2022). Additionally, the active metabolite 5-fluorouridine triphosphate (5-FUTP) is incorporated into RNA in place of uridine triphosphate, impairing transcriptions and protein synthesis in malignant cells (Hussain, 2020). Owing to the tumor microenvironment-dependent activation mechanism, the off-target cytotoxicity of capecitabine in normal cells is largely reduced (Alzahrani et al., 2023).

Since the approval by the United States Food and Drug Administration (FDA) for metastatic colorectal cancer in 1998, clinical applications of capecitabine have continued to expand (Yang et al., 2024). The combination of capecitabine and oxaliplatin (XELOX) is recommended in the United States NCCN Clinical Practice Guidelines in Oncology as first-line treatment for progressive gastric cancer (GC) (Ajani et al., 2022). Similarly, the Chinese 2023 Clinical Guidelines for the Diagnosis and Treatment of GC explicitly recommend capecitabine for first-line treatment of metastatic advanced GC, and for adjuvant chemotherapy following curative surgery in stage II-III GC patients (Wang et al., 2024a). While capecitabine has demonstrated proven efficacy in GC treatment, the safety concerns persist. Accumulating evidence indicates that capecitabine is highly associated with adverse events (AEs) including gastrointestinal disorders, hand-foot syndrome (HFS), hyperbilirubinemia, and diarrhea (Hagiwara et al., 2022; Santhosh et al., 2024; Nascimento et al., 2023). In addition, recent studies have documented some rare AEs outside of drug labeling, such as intestinal obstruction, penetrating aortic ulcer in cardiac disorders (Pow-Anpongkul et al., 2019; Liu et al., 2024). As the number of patients receiving capecitabine continues to increase, awareness of its safety profile remains insufficient among clinicians and patients. However, clinical trials are generally conducted under controlled conditions with strict eligibility criteria, which may limit the detection of rare, delayed, or treatment regimen-specific AEs. Given the increasingly complex safety challenges posed by capecitabine in clinical settings, there is a compelling need for related pharmacovigilance studies.

Using extensive datasets from the United States FDA AE Reporting System (FAERS), this study aims to comprehensively analyze the AE profile associated with capecitabine in GC therapy. Through validation of established risks and detection of novel safety signals, the study will inform the clinical optimization of treatment paradigms.

## 2 Materials and methods

### 2.1 Data sources

FAERS is one of the largest post-marketing drug safety surveillance databases worldwide and comprises AE case reports from 1969 to the present. This database is composed of seven datasets: AE coding (REAC), reported drug information (DRUG), patient demographics (DEMO), AE outcomes (OUTC), report source (RPSR), drug therapy timeline (THER), and drug use indications (INDI). As this study utilized fully de-identified public data, it was exempt from additional ethical review requirements.

### 2.2 Data processing

This study used “capecitabine” as the target drug and identified GC reports from the indication (INDI) file using the term “gastric cancer” (Sha et al., 2025). AE reports filed between 2004 Q1 and 2024 Q4. Subsequently, AE reports were stratified into four exclusive cohorts. Cohort 1 comprised AEs attributed to capecitabine monotherapy. Cohort 2 encompassed AEs associated with capecitabine in combination with other chemotherapeutic agents. Cohort 3 consisted of AEs linked to capecitabine in combination with other chemotherapeutic and targeted agents. Finally, Cohort 4 comprised AEs reported from capecitabine in combination with other chemotherapeutic agents as well as immunosuppressants.

**FIGURE 1:**
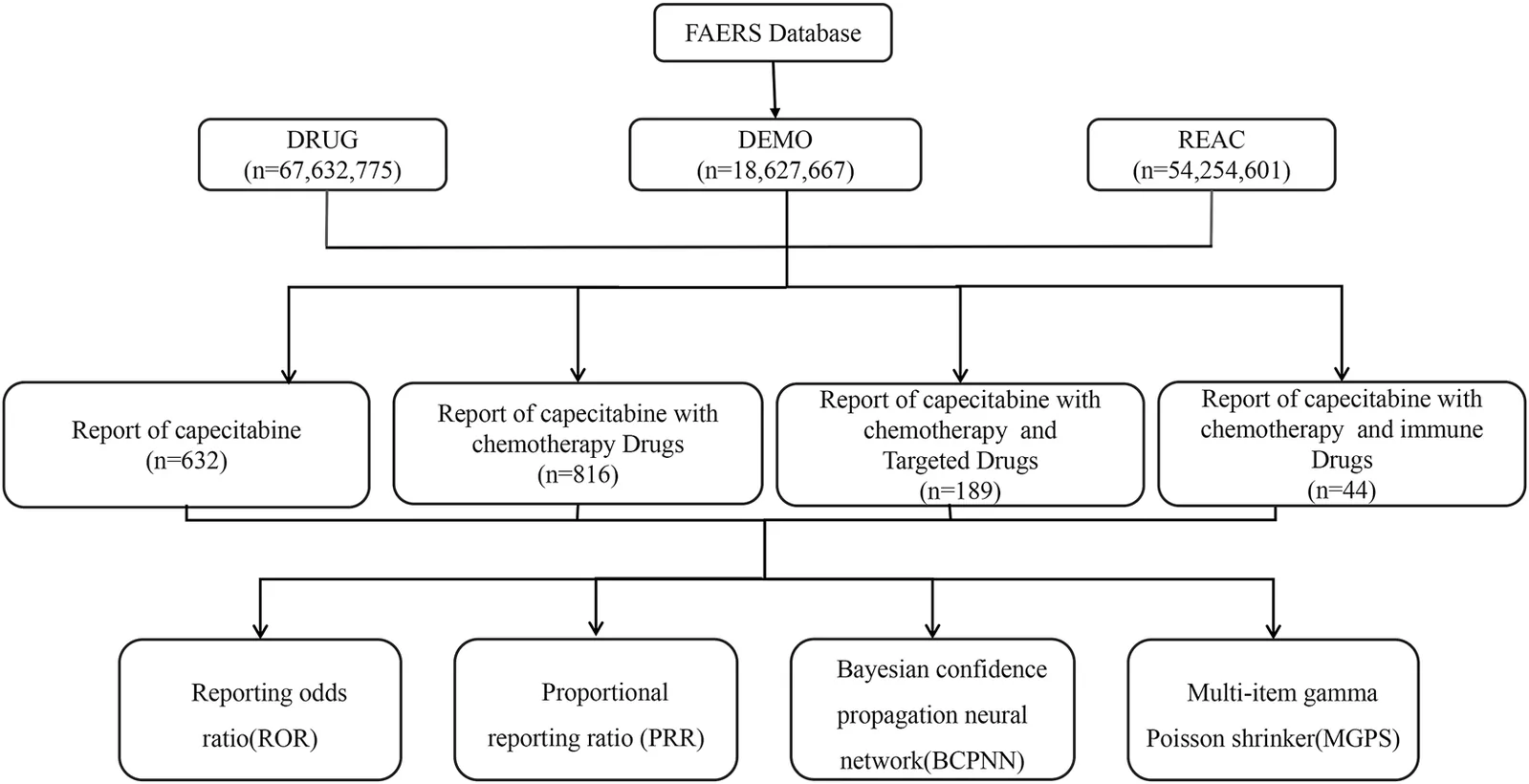
The flowchart for this study. FAERS, the United States Food and Drug Administration AE Reporting System; DRUG, reported drug information; DEMO, patients demographics; REAC, coding of adverse events.

Duplicate reports were removed according to the FDA-recommended deduplication strategy. When multiple reports shared the same CASEID, only the most recent record based on the FDA_DT was retained for analysis. AE reports in the FAERS system are classified into four categories: PS (primary suspected), SS (secondary suspected), C (concomitant), and I (interaction). To improve the specificity of signal detection and reduce potential confounding from concomitant medications, only reports in which capecitabine was classified as Primary Suspect (PS) were retained. Reports containing missing or inaccurate patient demographics or AE records were excluded. Standardization of the extracted AEs was performed using the system organ class (SOC) and preferred term (PT) from the Medical Dictionary for Regulatory Activities (MedDRA, version 26.0). SOC represents the highest level of MedDRA classification and categorizes adverse events according to organ systems or clinically relevant domains, whereas PT represents standardized specific medical concepts used to describe individual adverse events and serves as the primary level for signal detection and analysis. The study workflow is presented in Figure 1.

### 2.3 Statistical analysis

Descriptive analysis was used to present patient characteristics in reports of AEs, with results expressed as frequencies and percentages (%). Disproportionate analysis was applied to identify positive signals associated with SOC and PT linked to capecitabine, and to evaluate the association between AEs and capecitabine. Based on the 2 × 2 contingency table (Supplementary Table S1), this study utilized four integrated algorithms: including report odds ratio (ROR), proportion report ratio (PRR), bayesian confidence propagation neural network (BCPNN), and multi-item gamma poisson shrinker (MGPS). Among these, ROR served as the principal criterion for evaluating positive signals. The specific formulas and detection thresholds for these algorithms are outlined in Supplementary Table S2. Data processing and analysis were conducted using Microsoft Excel (version 2019), and R (version 4.4.1). Data visualization was carried out using the “ggplot2” package in R. P < 0.05 was considered statistically significant.

## 3 Results

### 3.1 Demographic information on the AE reports

Supplementary Table S3 indicates that 632 adverse event (AE) reports were ultimately assigned to Cohort 1 following data curation. Among these, 52.2% of AEs involved male patients; no instances involving minors were identified. The most frequent outcomes were death (24.5%), other medical events (21.5%) and hospitalization or prolonged hospitalization (20.7%). Most reports (61.2%) originated from the United States. Additionally, Cohorts 2, 3, and 4 comprised 816, 189, and 44 AE reports, respectively. Male patients accounted for 45.2%, 63.5%, and 38.6% of reports across Cohorts 2–4, respectively. The primary outcomes were other medical events (37.1%, 40.7%, and 40.9%), hospitalization or prolonged hospitalization (27.9%, 28.0% and 22.7%), and death (27.8%, 21.7%, and 29.5%). In Cohort 2, 52.2% of reports originated from other countries and regions. 39.7% of reports in Cohort 3 originated from Japan. In Cohort 4, the majority of reports originated from Others (41%), Japan (29.5%) and China (25.0%).

### 3.2 Analysis of AE signals at the PT level

After standardized coding, the 30 most common AE signals in each cohort were identified based on reporting frequency. AE signals at the PT level were analyzed using ROR, PRR, BCPNN, and MGPS. In Cohort 1 (Table 1), the three most common AEs were death (n = 83), diarrhea (n = 75), and nausea (n = 73); the three AEs with the strongest signals were palmar-plantar erythrodysaesthesia syndrome (n = 43, ROR = 4.62, 95% CI: 3.33–6.39), hypoaesthesia (n = 14, ROR = 3.45, 95% CI: 1.98–6.02), and intentional product use issue (n = 23, ROR = 3.26, 95% CI: 2.11–5.03).

**TABLE 1:** Signal intensity of Cohort 1 at preferred terms level.

PT	Counts	ROR (95% Cl)	PRR (*χ* ^*2*^)	EBGM (95% Cl)	IC (95% Cl)
Death	83	1.67 (1.33–2.1)	1.64 (20.27)	1.61 (1.33–1.94)	0.68 (−0.98–2.35)
Diarrhoea	75	1.71 (1.35–2.17)	1.68 (20.16)	1.65 (1.35–2.01)	0.72 (−0.95–2.39)
Nausea	73	1.93 (1.52–2.46)	1.89 (29.47)	1.84 (1.5–2.25)	0.88 (−0.79–2.55)
Off label use	59	1.34 (1.03–1.75)	1.33 (4.79)	1.32 (1.06–1.65)	0.4 (−1.27–2.07)
Vomiting	58	1.8 (1.38–2.36)	1.78 (18.91)	1.73 (1.38–2.17)	0.79 (−0.88–2.46)
Palmar-plantar erythrodysaesthesia syndrome	43	4.62 (3.33–6.39)	4.53 (102.76)	4.05 (3.08–5.31)	2.02 (0.34–3.69)
Disease progression	40	1.67 (1.21–2.3)	1.65 (9.92)	1.62 (1.24–2.12)	0.69 (−0.98–2.37)
Fatigue	40	2.09 (1.51–2.89)	2.06 (20.71)	1.99 (1.52–2.61)	0.99 (−0.68–2.67)
Decreased appetite	31	1.08 (0.75–1.56)	1.08 (0.19)	1.08 (0.8–1.46)	0.11 (−1.56–1.78)
Asthenia	25	1.39 (0.93–2.09)	1.39 (2.59)	1.37 (0.98–1.92)	0.45 (−1.22–2.12)
Intentional product use issue	23	3.26 (2.11–5.03)	3.23 (32.02)	3.01 (2.09–4.32)	1.59 (−0.09–3.27)
Malignant neoplasm progression	21	0.57 (0.37–0.88)	0.58 (6.53)	0.58 (0.41–0.84)	−0.77 (−2.44–0.9)
Weight decreased	18	2.23 (1.38–3.61)	2.22 (11.22)	2.13 (1.42–3.19)	1.09 (−0.59–2.77)
Gastric cancer	17	1.78 (1.09–2.92)	1.78 (5.47)	1.73 (1.15–2.61)	0.79 (−0.88–2.47)
Neuropathy peripheral	17	1.33 (0.82–2.17)	1.33 (1.34)	1.32 (0.87–1.98)	0.4 (−1.28–2.07)
Anaemia	16	0.65 (0.39–1.07)	0.65 (2.94)	0.66 (0.44–1)	−0.6 (−2.27–1.07)
Abdominal pain	16	1.2 (0.73–1.98)	1.2 (0.5)	1.19 (0.78–1.81)	0.25 (−1.42–1.92)
Thrombocytopenia	16	0.94 (0.57–1.55)	0.94 (0.05)	0.94 (0.62–1.44)	−0.08 (−1.75–1.59)
Neutropenia	16	0.64 (0.39–1.05)	0.64 (3.2)	0.65 (0.43–0.98)	−0.63 (−2.3–1.05)
Dehydration	15	1.17 (0.7–1.96)	1.17 (0.35)	1.16 (0.75–1.79)	0.21 (−1.46–1.89)
Hypoaesthesia	14	3.45 (1.98–6.02)	3.43 (21.65)	3.18 (1.99–5.06)	1.67 (−0.02–3.35)
Dysphagia	14	3.13 (1.8–5.45)	3.12 (18.22)	2.91 (1.83–4.63)	1.54 (−0.14–3.23)
Malaise	14	0.98 (0.57–1.67)	0.98 (0.01)	0.98 (0.62–1.53)	−0.03 (−1.71–1.64)
Leukopenia	14	1.19 (0.69–2.03)	1.18 (0.39)	1.18 (0.75–1.84)	0.24 (−1.44–1.91)
Mucosal inflammation	14	1.77 (1.03–3.04)	1.76 (4.37)	1.72 (1.09–2.7)	0.78 (−0.9–2.46)
Drug ineffective	14	2.08 (1.2–3.58)	2.07 (7.23)	2 (1.27–3.15)	1 (−0.68–2.68)
Stomatitis	13	1.35 (0.77–2.35)	1.34 (1.1)	1.33 (0.83–2.12)	0.41 (−1.27–2.09)
Rash	12	1.61 (0.9–2.89)	1.61 (2.62)	1.58 (0.97–2.57)	0.66 (−1.02–2.33)
Abdominal pain upper	12	2.21 (1.22–3.98)	2.2 (7.31)	2.11 (1.29–3.46)	1.08 (−0.6–2.76)
Constipation	11	1.55 (0.84–2.85)	1.55 (2.02)	1.52 (0.91–2.53)	0.6 (−1.08–2.28)

PT, preferred terms; ROR, reporting odds ratio; PRR, proportional reporting ratio; EBGM, empirical bayesian geometric mean; IC, information component; χ^2^, chi-squared; 95% CI, 95% confidence interval.

In Cohort 2 (Table 2), the three most frequently reported AEs were diarrhoea (n = 160), vomiting (n = 146), and nausea (n = 143). The three AEs with the strongest signals were palmar-plantar erythrodysaesthesia syndrome (n = 95, ROR = 6.69, 95% CI: 5.25–8.53), gastrointestinal disorder (n = 29, ROR = 6.3, 95% CI: 4.08–9.72), and neurotoxicity (n = 48, ROR = 5.59, 95% CI: 4.01–7.79).

**TABLE 2:** Signal intensity of Cohort 2 at preferred terms level.

PT	Counts	ROR (95% Cl)	PRR (χ^2^)	EBGM (95% Cl)	IC (95% Cl)
Diarrhoea	160	2.09 (1.76–2.47)	2.03 (76.02)	1.91 (1.66–2.2)	0.93 (−0.73–2.6)
Vomiting	146	2.68 (2.24–3.21)	2.61 (126.01)	2.37 (2.04–2.76)	1.25 (−0.42–2.92)
Nausea	143	2.14 (1.79–2.56)	2.09 (73.21)	1.96 (1.69–2.27)	0.97 (−0.7–2.64)
Neutropenia	104	2.56 (2.07–3.16)	2.51 (82.07)	2.29 (1.92–2.74)	1.2 (−0.47–2.87)
Anaemia	99	2.46 (1.99–3.05)	2.42 (71.98)	2.22 (1.86–2.66)	1.15 (−0.52–2.82)
Palmar-plantar erythrodysaesthesia syndrome	95	6.69 (5.25–8.53)	6.52 (313.2)	4.87 (3.97–5.97)	2.28 (0.61–3.96)
Thrombocytopenia	88	3.28 (2.6–4.14)	3.21 (112.01)	2.83 (2.33–3.44)	1.5 (−0.17–3.17)
Febrile neutropenia	63	1.82 (1.4–2.37)	1.81 (20.48)	1.72 (1.38–2.14)	0.78 (−0.89–2.45)
Leukopenia	60	3.14 (2.38–4.16)	3.1 (71.55)	2.75 (2.17–3.47)	1.46 (−0.22–3.13)
Decreased appetite	58	1.11 (0.85–1.45)	1.11 (0.57)	1.1 (0.88–1.38)	0.14 (−1.53–1.81)
Fatigue	56	1.58 (1.2–2.09)	1.57 (10.71)	1.52 (1.2–1.92)	0.6 (−1.07–2.27)
Death	54	0.55 (0.42–0.72)	0.55 (19.35)	0.57 (0.45–0.72)	−0.81 (−2.48–0.85)
Stomatitis	51	3.26 (2.41–4.42)	3.23 (65.03)	2.84 (2.2–3.66)	1.5 (−0.17–3.18)
Neurotoxicity	48	5.59 (4.01–7.79)	5.52 (131.1)	4.32 (3.27–5.71)	2.11 (0.43–3.79)
Mucosal inflammation	40	3.03 (2.15–4.25)	3 (44.82)	2.67 (2.01–3.55)	1.42 (−0.26–3.09)
Hepatic function abnormal	40	2.78 (1.98–3.9)	2.76 (38.15)	2.49 (1.87–3.3)	1.32 (−0.36–2.99)
Disease progression	38	0.83 (0.59–1.15)	0.83 (1.3)	0.84 (0.64–1.1)	−0.26 (−1.93–1.41)
Bone marrow failure	36	2.88 (2.01–4.11)	2.85 (36.72)	2.56 (1.9–3.46)	1.36 (−0.32–3.04)
White blood cell count decreased	35	1.56 (1.1–2.21)	1.55 (6.29)	1.5 (1.12–2.01)	0.59 (−1.09–2.26)
Dehydration	34	1.48 (1.04–2.11)	1.47 (4.76)	1.43 (1.07–1.93)	0.52 (−1.15–2.19)
Asthenia	33	0.98 (0.69–1.4)	0.98 (0.01)	0.98 (0.73–1.32)	−0.02 (−1.69–1.65)
Neuropathy peripheral	32	1.38 (0.96–1.99)	1.38 (3.09)	1.35 (0.99–1.83)	0.43 (−1.24–2.1)
General physical health deterioration	30	1.87 (1.28–2.73)	1.86 (10.67)	1.77 (1.28–2.43)	0.82 (−0.86–2.5)
Abdominal pain	29	1.19 (0.81–1.74)	1.19 (0.8)	1.17 (0.85–1.61)	0.23 (−1.44–1.9)
Gastrointestinal disorder	29	6.3 (4.08–9.72)	6.25 (90.99)	4.73 (3.29–6.79)	2.24 (0.55–3.93)
Platelet count decreased	29	1.14 (0.78–1.66)	1.14 (0.45)	1.13 (0.82–1.55)	0.17 (−1.5–1.85)
Renal impairment	28	2.61 (1.75–3.9)	2.6 (23.59)	2.37 (1.69–3.31)	1.24 (−0.44–2.92)
Pyrexia	25	0.64 (0.43–0.95)	0.64 (4.89)	0.66 (0.47–0.92)	−0.61 (−2.28–1.06)
Malaise	23	0.87 (0.57–1.32)	0.87 (0.45)	0.87 (0.61–1.24)	−0.19 (−1.87–1.48)
Neutrophil count decreased	23	0.99 (0.65–1.51)	0.99 (0)	0.99 (0.69–1.41)	−0.02 (−1.69–1.65)

PT, preferred terms; ROR, reporting odds ratio; PRR, proportional reporting ratio; EBGM, empirical bayesian geometric mean; IC, information component; χ^2^, chi-squared; 95% CI, 95% confidence interval.

In Cohort 3 (Table 3), the three most common AEs were diarrhoea (n = 32), nausea (n = 20), and palmar-plantar erythrodysaesthesia syndrome (n = 19). The three AEs with the strongest signals were leukoencephalopathy (n = 7, ROR = 21.79, 95% CI: 9.43–50.4), hepatotoxicity (n = 12, ROR = 17.79, 95% CI: 9.48–33.39), and toxicity to various agents (n = 9, ROR = 8.2, 95% CI: 4.11–16.35).

**TABLE 3:** Signal intensity of Cohort 3 at preferred terms level.

PT	Counts	ROR (*95% Cl*)	PRR (*χ* ^*2*^)	EBGM (*95% Cl*)	IC (*95% Cl*)
Diarrhoea	32	1.97 (1.38-2.82)	1.92 (14.22)	1.9 (1.41-2.57)	0.93 (-0.74-2.6)
Nausea	20	1.38 (0.88-2.16)	1.37 (2.01)	1.36 (0.94-1.98)	0.45 (-1.22-2.12)
Palmar-plantar erythrodysaesthesia syndrome	19	5.22 (3.26-8.35)	5.09 (59.08)	4.85 (3.27-7.18)	2.28 (0.6-3.95)
Neutropenia	14	1.56 (0.91-2.66)	1.55 (2.69)	1.54 (0.98-2.4)	0.62 (-1.05-2.29)
Vomiting	13	1.05 (0.61-1.83)	1.05 (0.03)	1.05 (0.66-1.67)	0.07 (-1.6-1.74)
Decreased appetite	13	1.23 (0.71-2.15)	1.23 (0.56)	1.23 (0.77-1.95)	0.29 (-1.38-1.97)
Hepatotoxicity	12	17.79 (9.48-33.39)	17.48 (153.23)	14.53 (8.58-24.6)	3.86 (2.15-5.57)
Fatigue	11	1.5 (0.82-2.74)	1.49 (1.78)	1.48 (0.9-2.46)	0.57 (-1.1-2.24)
Mucosal inflammation	11	3.83 (2.08-7.05)	3.78 (21.6)	3.66 (2.2-6.09)	1.87 (0.19-3.55)
Anaemia	11	1.24 (0.68-2.25)	1.23 (0.48)	1.23 (0.74-2.03)	0.3 (-1.38-1.97)
Pyrexia	10	1.31 (0.7-2.47)	1.31 (0.73)	1.3 (0.77-2.21)	0.38 (-1.29-2.06)
Bone marrow failure	10	3.7 (1.95-7.01)	3.66 (18.55)	3.54 (2.08-6.04)	1.82 (0.14-3.5)
Febrile neutropenia	9	1.23 (0.63-2.39)	1.23 (0.37)	1.22 (0.7-2.13)	0.29 (-1.38-1.96)
Pneumonia	9	1.84 (0.94-3.57)	1.83 (3.31)	1.81 (1.04-3.15)	0.85 (-0.82-2.53)
Hepatic function abnormal	9	2.88 (1.47-5.62)	2.85 (10.49)	2.79 (1.59-4.88)	1.48 (-0.2-3.16)
Toxicity to various agents	9	8.2 (4.11-16.35)	8.1 (50.97)	7.45 (4.18-13.27)	2.9 (1.2-4.59)
Disease progression	9	0.99 (0.51-1.91)	0.99 (0)	0.99 (0.57-1.72)	-0.02 (-1.69-1.65)
Asthenia	8	1.19 (0.59-2.41)	1.19 (0.24)	1.19 (0.66-2.14)	0.25 (-1.43-1.92)
Myelosuppression	8	1.43 (0.71-2.9)	1.43 (1.01)	1.42 (0.79-2.56)	0.51 (-1.17-2.18)
Cerebral infarction	7	4.6 (2.14-9.89)	4.56 (18.45)	4.37 (2.3-8.29)	2.13 (0.44-3.82)
Leukoencephalopathy	7	21.79 (9.43-50.4)	21.57 (108.24)	17.2 (8.53-34.7)	4.1 (2.36-5.85)
Platelet count decreased	7	1.37 (0.64-2.89)	1.36 (0.66)	1.36 (0.72-2.54)	0.44 (-1.24-2.11)
Stomatitis	7	1.97 (0.93-4.19)	1.96 (3.23)	1.94 (1.03-3.64)	0.95 (-0.72-2.63)
Leukopenia	7	1.61 (0.76-3.42)	1.61 (1.58)	1.59 (0.85-2.99)	0.67 (-1-2.35)
Pancytopenia	7	5.19 (2.41-11.19)	5.14 (22.01)	4.89 (2.57-9.31)	2.29 (0.6-3.98)
Pulmonary embolism	6	2.1 (0.93-4.74)	2.09 (3.34)	2.06 (1.04-4.08)	1.04 (-0.64-2.72)
Gastric cancer	6	1.68 (0.74-3.77)	1.67 (1.58)	1.66 (0.84-3.27)	0.73 (-0.95-2.41)
Neuropathy peripheral	6	1.26 (0.56-2.84)	1.26 (0.32)	1.26 (0.64-2.48)	0.33 (-1.35-2.01)
White blood cell count decreased	6	1.29 (0.57-2.9)	1.29 (0.38)	1.28 (0.65-2.52)	0.36 (-1.32-2.03)
Weight decreased	6	1.95 (0.87-4.41)	1.95 (2.71)	1.92 (0.97-3.8)	0.94 (-0.74-2.62)

The red markings indicate positive signals. PT, preferred terms; ROR, reporting odds ratio; PRR, proportional reporting ratio; EBGM, empirical bayesian geometric mean; IC, information component; χ^2^, chi-squared; 95% CI, 95% confidence interval.

All reports from Cohort 4 were low in number (Table 4), and the four most frequent AEs were decreased appetite (n = 8), malignant neoplasm progression (n = 7), fatigue (n = 7), and malaise (n = 7). The three AEs with the strongest signals were sleep disorder (n = 5, ROR = 95.98, 95% CI: 33.54–274.69), dry mouth (n = 5, ROR = 35.98, 95% CI: 13.89–93.18), and inappropriate schedule of product administration (n = 5, ROR = 28.78, 95% CI: 11.25–73.59).

**TABLE 4:** Signal intensity of Cohort 4 at preferred terms level.

PT	Counts	ROR (95% Cl)	PRR (χ^2^)	EBGM (95% Cl)	IC (95% Cl)
Decreased appetite	8	2.16 (1.06-4.38)	2.12 (4.75)	2.11 (1.16-3.81)	1.07 (-0.6-2.75)
Malignant neoplasm progression	7	1.49 (0.7-3.17)	1.48 (1.1)	1.47 (0.78-2.77)	0.56 (-1.12-2.24)
Fatigue	7	2.71 (1.27-5.77)	2.66 (7.23)	2.64 (1.4-4.97)	1.4 (-0.28-3.08)
Malaise	7	3.82 (1.79-8.17)	3.74 (13.93)	3.69 (1.96-6.97)	1.89 (0.2-3.57)
Asthenia	6	2.54 (1.12-5.74)	2.5 (5.4)	2.48 (1.26-4.91)	1.31 (-0.37-2.99)
Death	6	0.87 (0.39-1.97)	0.88 (0.11)	0.88 (0.44-1.73)	-0.19 (-1.87-1.49)
Disease progression	5	1.55 (0.64-3.76)	1.53 (0.93)	1.53 (0.73-3.22)	0.61 (-1.07-2.29)
Pain	5	6.11 (2.49-14.98)	5.99 (20.34)	5.87 (2.77-12.43)	2.55 (0.86-4.24)
Condition aggravated	5	8.91 (3.61-21.97)	8.74 (33.06)	8.45 (3.97-17.98)	3.08 (1.38-4.77)
Anxiety	5	23.49 (9.27-59.5)	23 (95.57)	20.96 (9.63-45.63)	4.39 (2.67-6.11)
Inappropriate schedule of product administration	5	28.78 (11.25-73.59)	28.17 (116.59)	25.16 (11.47-55.18)	4.65 (2.92-6.38)
Dry mouth	5	35.98 (13.89-93.18)	35.22 (143.87)	30.59 (13.8-67.83)	4.94 (3.19-6.68)
Sleep disorder	5	95.98 (33.54-274.69)	93.92 (324.56)	66.59 (27.63-160.51)	6.06 (4.25-7.87)
Nausea	4	0.76 (0.28-2.04)	0.76 (0.31)	0.76 (0.33-1.74)	-0.39 (-2.07-1.28)
Neutropenia	4	1.23 (0.46-3.32)	1.23 (0.17)	1.23 (0.53-2.81)	0.29 (-1.39-1.97)
Thrombocytopenia	4	1.81 (0.67-4.87)	1.79 (1.4)	1.79 (0.78-4.1)	0.84 (-0.84-2.52)
Ascites	4	2.76 (1.02-7.47)	2.73 (4.37)	2.71 (1.18-6.23)	1.44 (-0.24-3.12)
Metastases to liver	3	6.57 (2.07-20.87)	6.5 (13.6)	6.35 (2.41-16.69)	2.67 (0.97-4.37)
Vomiting	3	0.67 (0.21-2.1)	0.68 (0.47)	0.68 (0.26-1.76)	-0.56 (-2.24-1.12)
Anaemia	3	0.93 (0.3-2.93)	0.94 (0.01)	0.94 (0.36-2.43)	-0.1 (-1.78-1.58)
Colitis	3	8.66 (2.71-27.63)	8.56 (19.33)	8.28 (3.14-21.87)	3.05 (1.34-4.76)
Diarrhoea	3	0.49 (0.16-1.53)	0.5 (1.57)	0.5 (0.19-1.29)	-1.01 (-2.69-0.67)
Infusion related reaction	3	5.84 (1.84-18.52)	5.78 (11.59)	5.66 (2.16-14.86)	2.5 (0.8-4.2)
Type 1 diabetes mellitus	3	16.7 (5.13-54.31)	16.49 (40.72)	15.44 (5.75-41.42)	3.95 (2.22-5.68)
Neuropathy peripheral	3	1.77 (0.56-5.56)	1.76 (0.99)	1.76 (0.67-4.57)	0.81 (-0.87-2.49)
Neutrophil count decreased	3	1.8 (0.57-5.66)	1.79 (1.05)	1.79 (0.69-4.65)	0.84 (-0.85-2.52)
Pneumonitis	3	6.16 (1.94-19.53)	6.09 (12.46)	5.96 (2.27-15.65)	2.57 (0.88-4.27)
White blood cell count decreased	3	1.8 (0.57-5.66)	1.79 (1.05)	1.79 (0.69-4.65)	0.84 (-0.85-2.52)
Drug hypersensitivity	3	20.14 (6.14-66.04)	19.89 (49.49)	18.36 (6.8-49.59)	4.2 (2.46-5.94)
Off label use	2	0.33 (0.08-1.33)	0.34 (2.68)	0.34 (0.11-1.08)	-1.57 (-3.24-0.11)

The red markings indicate positive signals. PT, preferred terms; ROR, reporting odds ratio; PRR, proportional reporting ratio; EBGM, empirical bayesian geometric mean; IC, information component; χ^2^, chi-squared; 95% CI, 95% confidence interval.

### 3.3 Relationship between main AE signals and SOCs

**FIGURE 2:**
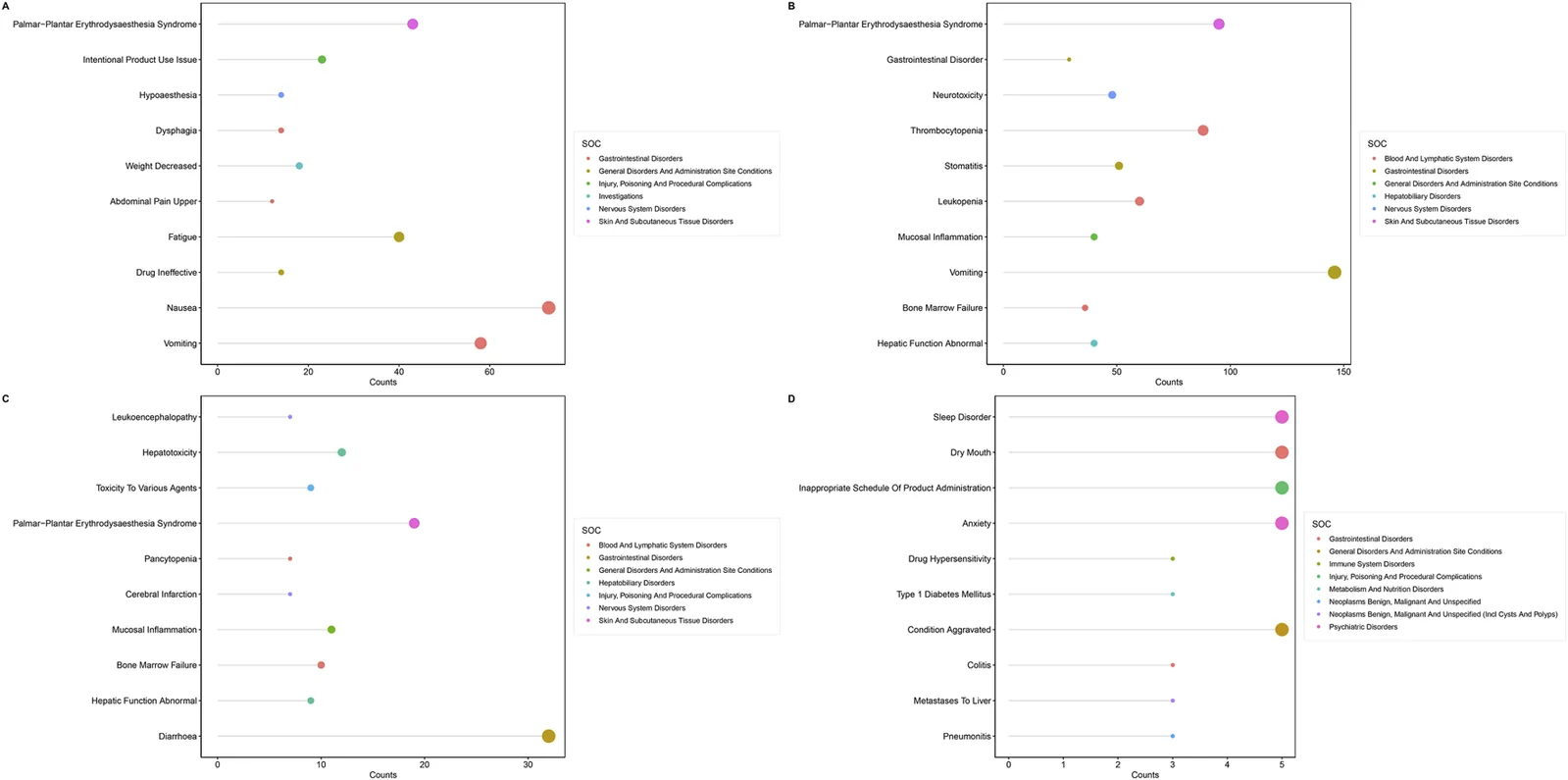
The corresponding SOCs of the key PT signals. SOC: system organ class; **(A)** Cohort 1, including capecitabine monotherapy; **(B)** Cohort 2, including capecitabine co-administered with other chemotherapeutic agents; **(C)** Cohort 3, including capecitabine in combination with other chemotherapeutic and targeted agents; **(D)** Cohort 4, including capecitabine plus chemotherapeutic agents as well as immunosuppressants.

The AE signals of the four cohorts were categorized according to SOC. Subsequently, the top 10 PTs in terms of signal intensity and their corresponding SOCs for each cohort were visually analyzed. The top 10 AEs ranked by signal strength in the Cohort 2 exhibited distinct composition compared to Cohort 1 (Figure 2A), including gastrointestinal disorder, neurotoxicity, thrombocytopenia, stomatitis, leukopenia, mucosal inflammation, bone marrow failure, and hepatic function abnormality (Figure 2B).

Different AEs were present in the top 10 signal intensity rankings in Cohort 3 compared to Cohort 1, including leukoencephalopathy, hepatotoxicity, toxicity to various agents, pancytopenia, cerebral infarction, mucosal inflammation, bone marrow failure, hepatic function abnormality, and diarrhoea (Figure 2C).

It is worth noting that the top 10 AEs in Cohort 4 in terms of signal strength were completely different from those of Cohort 1, in the order of sleep disorder, dry mouth, inappropriate schedule of product administration, anxiety, drug hypersensitivity, type 1 diabetes mellitus, condition aggravated, colitis, metastases to liver, and pneumonitis (Figure 2D).

Furthermore, the results showed that gastrointestinal disorders and general disorders and administration site conditions were common SOCs across all four cohorts. Compared with Cohort 1, blood and lymphatic system disorders and hepatobiliary disorders were newly identified in Cohorts 2 and 3 (Figures 2B,C). In Cohort 4, additional SOCs not observed in Cohort 1 included psychiatric disorders, immune system disorders, metabolism and nutrition disorders, neoplasms benign, malignant and unspecified (including cysts and polyps), and neoplasms benign, malignant and unspecified (Figure 2D).

### 3.4 The occurrence time of AE

**FIGURE 3:**
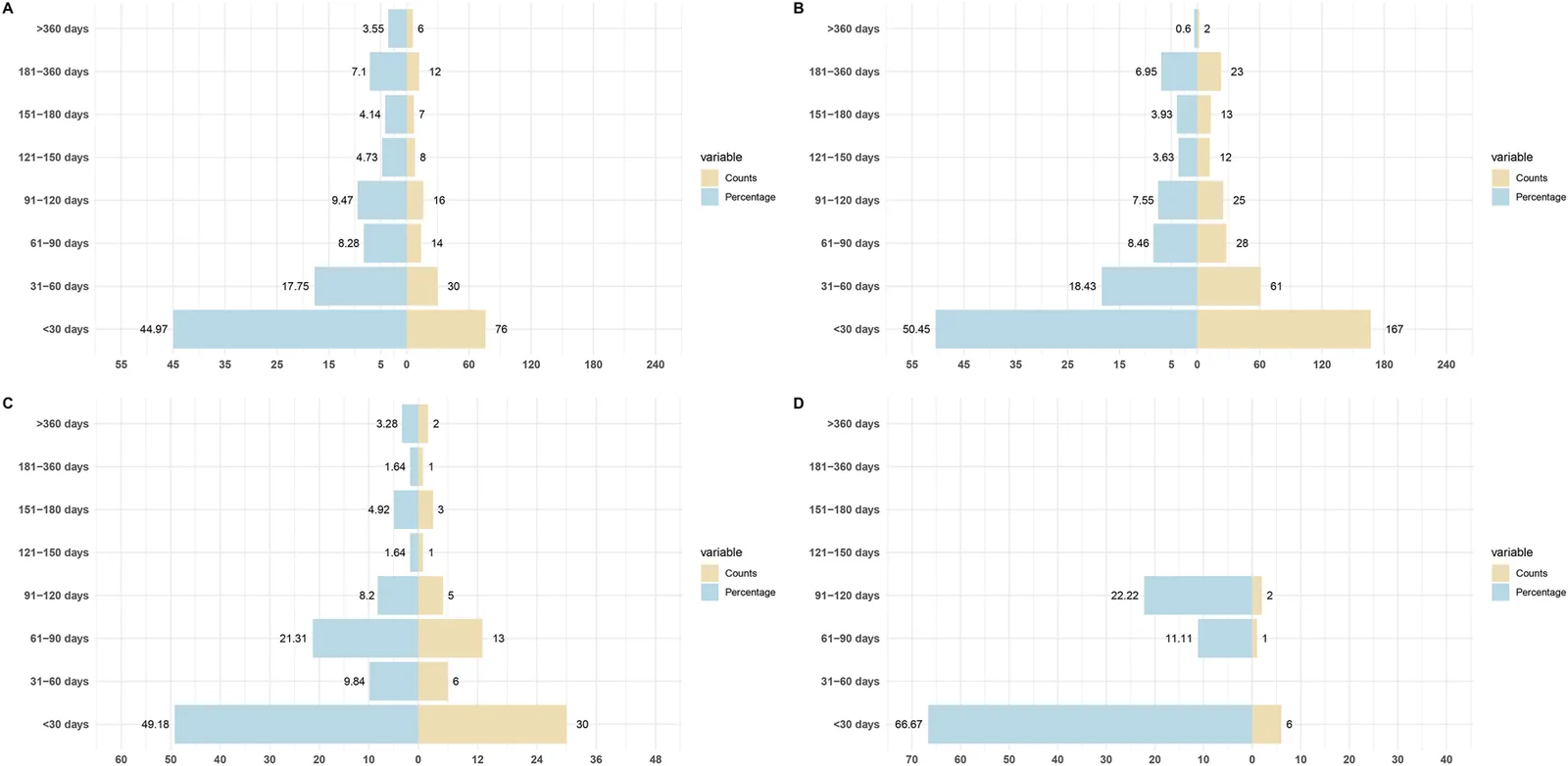
The occurrence time of capecitabine-based AEs. **(A)** Cohort 1, including capecitabine monotherapy; **(B)** Cohort 2, including capecitabine co-administered with other chemotherapeutic agents; **(C)** Cohort 3, including capecitabine in combination with other chemotherapeutic and targeted agents; **(D)** Cohort 4, including capecitabine plus chemotherapeutic agents as well as immunosuppressants.

In all four cohorts, the majority of AEs occurred within 30 days of medication initiation (Figure 3). In Cohort 1, the overall incidence of AEs showed a downward trend, but there was a slight increase in the number of AE occurrences between 91–120 days and 181–360 days. In Cohort 2, the overall incidence of AEs showed a downward trend, but the number and incidence of AEs from 181 to 360 days rebounded. In Cohort 3, AEs from 121 to 150 days and 181–360 days were the least frequent.

## 4 Discussion

Previously reported safety events associated with capecitabine primarily stem from clinical trial studies and individual case reports. Owing to the different designs of clinical trials and the strict inclusion criteria, rare AEs may be underreported. This study focused on AEs associated with capecitabine treatment for GC by analyzing data from the FAERS database spanning from 2004 Q1 to 2024 Q4. After validation using four mismatch analysis algorithms, the relevant safety signals were identified.

Across all four cohorts, male patients accounted for a higher proportion of reports than female patients, while older adults accounted for a higher proportion of reports than younger adults. These demographic characteristics are likely to reflect the distribution of patients receiving capecitabine and the epidemiology of GC rather than indicating an increased susceptibility to AEs (Lin et al., 2024). Geographically, the majority of reports originated from East Asia and the United States. The distribution likely reflectes elevated incidence of GC and the robust pharmacovigilance infrastructure in these regions. It is worth noting that dihydropyrimidine dehydrogenase (DPD) catalyses the initial and rate-limiting step in 5-FU catabolism (Forouzesh and Moran, 2021). Given the high prevalence of DPD gene polymorphisms among Asian populations, pre-treatment genotyping is recommended (Chan et al., 2024; Hishinuma et al., 2022; Biagioni et al., 2020). Patients harbouring loss-of-function variants should receive an empirically reduced capecitabine dose and alternative regimens when clinically indicated.

Furthermore, the findings of this real-world pharmacovigilance study highlight the distinct safety profiles of capecitabine across different treatment regimens in GC patients. First, the two highest-weight SOCs, gastrointestinal disorders and general disorders and administration site conditions were consistently observed across all four cohorts. It should be noted that, in the MedDRA terminology, the SOC “General disorders and administration site conditions” does not specifically refer to injection or topical application sites. Instead, this broad SOC encompasses a variety of general clinical conditions reported during drug treatment, including systemic symptoms such as fatigue, asthenia, and pyrexia. For orally administered capecitabine, the identification of this SOC should therefore not be interpreted as evidence of administration-site toxicity, but rather as a reflection of general treatment-related clinical manifestations categorized according to the MedDRA hierarchy. With the exception of the cohort receiving concomitant chemotherapy and immunosuppressive agents, the pronounced signal of palmar-plantar erythrodysaesthesia syndrome warrants attention. Overall, the profile of AEs is consistent with the known safety spectrum of oral fluoropyrimidines, dominated by gastrointestinal and skin toxicity. Multiple pieces of evidence indicate that capecitabine-related AEs most frequently involve gastrointestinal toxicity and HFS (Liu et al., 2024; Qadar et al., 2024; Pan et al., 2024a). Chen et al. reported that the AE signals associated with capecitabine were primarily concentrated in gastrointestinal disorders, skin and subcutaneous tissue disorders, general disorders and administration site conditions, and blood and lymphatic system disorders (Chen et al., 2022). This observation is attributable to 5-FU-mediated disruption of DNA synthesis and its pronounced cytotoxicity toward rapidly proliferating gastrointestinal mucosal and epidermal keratinocyte populations (Liu et al., 2024; Wang et al., 2024b).

Compared with monotherapy, the combination of other chemotherapeutic and targeted agents markedly increases the incidence of hepatobiliary disorders and blood and lymphatic system disorders. Extensive evidence indicates that cytotoxic agents directly impair hematopoietic stem cells (Feng et al., 2022). Concurrent use of capecitabine with platinum- and taxane-based agents intensifies bone marrow suppression and neurotoxicity, with possibly hepatic damage (Kawabata et al., 2023; Mayer et al., 2021). Likewise, regimens combining anti-angiogenic agents with chemotherapy increase the risk of hematologic or hepatic toxicity relative to monotherapy (Xie and Zhou, 2024). Therefore, patients receiving these regimens require regular monitoring of hematologic and hepatic parameters, and supportive therapy or dose modifications should be implemented as clinically indicated.

Notably, although the triple combination of capecitabine, platinum-based agents, and targeted agents has demonstrated substantial antitumor efficacy, its neurotoxicity warrants vigilant monitoring. Previous pharmacovigilance studies of oxaliplatin have also reported neurotoxicity-related safety signals, which are consistent with and support our findings (Pan et al., 2024b). A previous pharmacovigilance study based on the French Pharmacovigilance Database also identified leukoencephalopathy as a rare neurological AE associated with fluoropyrimidines, including capecitabine, which is consistent with our findings (Perrain et al., 2021). Clinical reports indicate that patients receiving capecitabine, oxaliplatin, and bevacizumab for metastatic colorectal cancer may develop dysarthria, ataxia, and MRI-confirmed multifocal leukoencephalopathy (Bougea et al., 2016). The use of bevacizumab is associated with an elevated risk of cerebrovascular events, particularly among patients with uncontrolled hypertension (Zuo et al., 2014). Although these severe neurological AEs are relatively rare, they can be life-threatening or disabling. Consequently, when triple therapy is used as a first-line treatment regimen, blood pressure and coagulation status must be strictly controlled.

Moreover, this study identified potential signals involving psychiatric, immune, and metabolic disorders in Cohort 4. These signals were based on a very limited number of reports (≤5 cases), and their statistical significance may have been influenced by the rarity of the events and reporting bias inherent to the FAERS database. Therefore, these findings should be interpreted with caution. Furthermore, because this study was based on spontaneous reports from the FAERS database and all patients received combination therapy, it was not possible to distinguish the specific contribution of capecitabine from that of concomitant medications to these AEs. Accordingly, these findings should be regarded as preliminary safety signals requiring further validation rather than established treatment-related toxicities, and should be confirmed in large-scale pharmacoepidemiological studies and prospective clinical investigations.

Importantly, the safety signals identified in this study should be interpreted by distinguishing established toxicities from potential emerging signals. Consistent with previous clinical trials (Liu et al., 2024) and post-marketing surveillance studies, gastrointestinal disorders, and general disorders and administration site conditions represented well-recognized toxicities associated with capecitabine and were consistently detected across different treatment cohorts. These findings further support the ability of FAERS-based pharmacovigilance analyses to capture the established safety profile of capecitabine. In contrast, several signals observed in specific combination regimens may provide additional insights into the safety profile of capecitabine in real-world clinical practice. For example, the increased signals of hepatobiliary disorders observed in patients receiving capecitabine combined with chemotherapy and targeted agents may reflect potential additive toxicity from multi-agent regimens. Furthermore, rare neurological signals, including leukoencephalopathy, highlight the importance of clinical vigilance when capecitabine is administered with neurotoxic agents such as platinum-based chemotherapy. However, these findings should be considered potential emerging safety signals rather than confirmed toxicities, because spontaneous reporting databases cannot establish causality.

The time-to-onset analysis showed that most AEs occurred within the first 30 days after treatment initiation, suggesting that close clinical monitoring during the early phase of capecitabine therapy may facilitate the timely identification and management of AEs. This finding is clinically relevant because early recognition of gastrointestinal toxicity, hand-foot syndrome, and hematological abnormalities may enable prompt supportive interventions or dose adjustments. However, these findings should be interpreted with caution. Time-to-onset estimates derived from the FAERS database may be influenced by incomplete or missing event dates, variability in treatment duration among patients, and potential confounding from concomitant medications or combination regimens. Therefore, the observed temporal patterns should be considered descriptive and require confirmation in prospective clinical studies.

Overall, our findings are broadly concordant with the existing literature on capecitabine safety across gastric and other malignancies. The common AEs associated with capecitabine were reaffirmed, whereas uncommon immune-mediated toxicities reflect the established safety profile of immune checkpoint inhibitors. Future investigations should elucidate the underlying mechanisms of these interactions.

This study has several limitations. First, as a spontaneous reporting system, the FAERS database is subject to underreporting, reporting bias, duplicate reporting, and incomplete clinical information, which may affect signal detection. Second, disproportionality analysis identifies statistical associations rather than causal relationships, and the detected signals should be interpreted with caution. Third, detailed information on drug dosage, treatment duration, disease severity, comorbidities, and concomitant medications was unavailable, precluding adjustment for potential confounding factors. Finally, the number of reports in Cohort 4 was relatively small, and several signals were based on only a few cases. Therefore, these findings should be considered preliminary and require further validation in large-scale pharmacoepidemiological studies and prospective clinical investigations.

## 5 Conclusion

This study comprehensively characterized the AE profiles associated with capecitabine-based treatment regimens for GC using the FAERS database. Gastrointestinal disorders and general disorders were the most consistently reported AEs, whereas combination regimens with cytotoxic, targeted, or immunotherapeutic agents exhibited distinct safety profiles, including additional hematologic, hepatobiliary, neurological, and potential immune-related AE signals. Most AEs occurred within the first 30 days after treatment initiation, highlighting the importance of early safety monitoring. As this study was based on spontaneous reporting data, the detected signals should be interpreted cautiously and require further validation in well-designed epidemiological and prospective clinical studies.

## Data Availability

The original contributions presented in the study are included in the article/Supplementary Material, further inquiries can be directed to the corresponding authors.
