# Supplementary material for: Adverse events of capecitabine in gastric cancer patients: a real‐world pharmacovigilance study based on the FAERS database
Source: Front Pharmacol. 2026 Sep 11;17:1861329. doi: 10.3389/fphar.2026.1861329 (PMC13612287; doi:10.3389/fphar.2026.1861329)
Supplement: Supplementary file 1 [file Table1.docx]

Supplementary Table S1. Two-by-two contingency table for disproportionality analyses

|  | Target adverse events | All other adverse events | Total |
| --- | --- | --- | --- |
| Target drug | a | b | a+b |
| All other drugs | c | d | c+d |
| Total | a+c | b+d | N=a+b+c+d |
