# Supplementary material for: Adverse events of capecitabine in gastric cancer patients: a real‐world pharmacovigilance study based on the FAERS database
Source: Front Pharmacol. 2026 Sep 11;17:1861329. doi: 10.3389/fphar.2026.1861329 (PMC13612287; doi:10.3389/fphar.2026.1861329)
Supplement: Supplementary file 2 [file Table2.docx]

Supplementary Table S2. Principle of disproportionality measure and standard of signal detection.

| Methods | Calculation formula | Inclusion standard of positive signal |
| --- | --- | --- |
| ROR | ROR = ad/bc | N ≥ 3, the lower limit of *95% CI* > 1 |
|  | *95% CI* = e^ln(ROR)±1.96(1/a+1/b+1/c+1/d)^0.5^ |  |
| PRR | PRR = [a(c+d)]/[c(a+b)] | PRR ≥ 2, *χ^2^* ≥ 4, N ≥ 3 |
|  | *χ^2^* =[(ad-bc)^2](a+b+c+d)/[(a+b)(c+d)(a+c)(b+d)] |  |
| BCPNN | IC = log_2_a(a+b+c+d)/[(a+c)(a+b)] | IC025 > 0 |
|  | *95% CI* = E(IC) ± 2[V(IC)]^0.5 |  |
| MGPS | EBGM = a(a+b+c+d)/[(a+c)(a+b)] | EBGM05 > 2 |
|  | 95% *CI* = e^ln(EBGM)±1.96(1/a+1/b+1/c+1/d)^0.5^ |  |

a: number of reports containing both the target drug and the target adverse drug reaction; b: number of reports containing other adverse drug reactions of the target drug; c: number of reports containing the target adverse drug reaction of other drugs; d: number of reports containing other drugs and other adverse drug reactions; N: total reports; ROR: reporting odds ratio; 95% CI: 95% confidence interval; PRR: proportional reporting ratio; χ^2^: chi-squared; BCPNN: bayesian confidence propagation neural network; IC: information component; E (IC): the IC expectations; V (IC): the variance of IC; IC025: the lower limit of the 95% CI of the IC; MGPS: multi-item gamma poisson shrinker; EBGM: empirical bayesian geometric mean; EBGM05: the one-sided 95% confidence lower bound for the EBGM.
