# Supplementary material for: Adverse events of capecitabine in gastric cancer patients: a real‐world pharmacovigilance study based on the FAERS database
Source: Front Pharmacol. 2026 Sep 11;17:1861329. doi: 10.3389/fphar.2026.1861329 (PMC13612287; doi:10.3389/fphar.2026.1861329)
Supplement: Supplementary file 3 [file Table3.docx]

Supplementary Table S3. Clinical characteristics of AE reports with capecitabine

| Variable | Cohort 1  (n = 632) | Cohort 2  (n = 816) | Cohort 3  (n = 189) | Cohort 4  (n = 44) |
| --- | --- | --- | --- | --- |
| Sex |  |  |  |  |
| Female | 233 (36.9%) | 164 (20.1%) | 30 (15.9%) | 13 (29.5%) |
| Male | 330 (52.2%) | 369 (45.2%) | 120 (63.5%) | 17 (38.6%) |
| Missing | 69 (10.9%) | 283 (34.7%) | 39 (20.6%) | 14 (31.8%) |
| Weight (kg) |  |  |  |  |
| < 50 | 23 (3.6%) | 42 (5.1%) | 12 (6.3%) | 1 (2.3%) |
| 50 - 100 | 146 (23.1%) | 203 (24.9%) | 41 (21.7%) | 9 (20.5%) |
| > 100 | 9 (1.4%) | 3 (0.4%) | 0 | 0 |
| Missing | 454 (71.8%) | 42 (5.1%) | 12 (6.3%) | 1 (2.3%) |
| Age (years) |  |  |  |  |
| ≤ 17 | 0 | 3 (0.4%) | 0 | 2 (4.5%) |
| 18 - 64 | 158 (25.0%) | 203 (24.9%) | 54 (28.6%) | 15 (34.1%) |
| 65 - 85 | 181 (28.6%) | 209 (25.6%) | 73 (38.6%) | 12 (27.3%) |
| ≥ 86 | 20 (3.2%) | 2 (0.2%) | 0 | 0 |
| Missing | 273 (43.2%) | 399 (48.9%) | 62 (32.8%) | 15 (34.1%) |
| Occupation of the reporter |  |  |  |  |
| Consumer | 172 (27.2%) | 19 (2.3%) | 4 (2.1%) | 0 |
| Health professional | 62 (9.8%) | 109 (13.4%) | 47 (24.9%) | 33 (75.0%) |
| Physician | 139 (22.0%) | 510 (62.5%) | 104 (55.0%) | 7 (15.9%) |
| Others | 103 (16.3%) | 150 (18.4%) | 16 (8.5%) | 1 (2.3%) |
| Pharmacist | 72 (11.4%) | 21 (2.6%) | 16 (8.5%) | 3 (6.8%) |
| Missing | 84 (13.3%) | 7 (0.9%) | 2 (1.1%) | 33 (75.0%) |
| Outcomes |  |  |  |  |
| Death | 155 (24.5%) | 227 (27.8%) | 41 (21.7%) | 13 (29.5%) |
| Disability | 5 (0.8%) | 2 (0.2%) | 2 (1.1%) | 0 |
| Hospitalization (initial or prolonged) | 131 (20.7%) | 228 (27.9%) | 53 (28.0%) | 10 (22.7%) |
| Life-threatening | 6 (0.9%) | 38 (4.7%) | 11 (5.8%) | 2 (4.5%) |
| Other medical events | 136 (21.5%) | 303 (37.1%) | 77 (40.7%) | 18 (40.9%) |
| Missing | 199 (31.5%) | 18 (2.2%) | 5 (2.6%) | 1 (2.3%) |
| Country |  |  |  |  |
| China | 51 (8.1%) | 190 (23.3%) | 41 (21.7%) | 11 (25.0%) |
| Japan | 17 (2.7%) | 114 (14.0%) | 75 (39.7%) | 13 (29.5%) |
| United States | 387 (61.2%) | 86 (10.5%) | 11 (5.8%) | 2 (4.5%) |
| Others | 177(28%) | 426(52.2%) | 62(32.8%) | 18(41%) |

Cohort 1: AEs attributed to capecitabine monotherapy; Cohort 2: AEs associated with capecitabine in combination with other chemotherapeutic agents; Cohort 3: AEs linked to capecitabine in combination with other chemotherapeutic and targeted agents; Cohort 4: AEs reported from capecitabine in combination with other chemotherapeutic agents as well as immunosuppressants.
